# Supplementary material for: Effects of 5-Aminolevulinic Acid as a Supplement on Animal Performance, Iron Status, and Immune Response in Farm Animals: A Review
Source: Animals (Basel). 2020 Aug 4;10(8):1352. doi: 10.3390/ani10081352 (PMC7459508; doi:10.3390/ani10081352)
Supplement: Supplementary file 1 [file animals-10-01352-s001.zip › Supplementary Files/Table S1.docx]

**Table S1.** Summary of the general data of papers evaluating the effect of 5-ALA supplementation on farm animals.

| **Species** | **Dose^1^** | **Duration^2^** | **Evaluated outcome measures** | **Results** | **Ref.** |
| --- | --- | --- | --- | --- | --- |
| Laying hens | 5 mg  10 mg  15 mg | 56 d (from 40 wk of age) | Egg production and quality e.g., egg (weight, shell breaking strength, shell thickness, yolk index, yolk color unit, and haugh unit); RBC, Hb, Fe, and TIBC; WBC, and lymphocyte | No differences in Hb, Fe, TIBC, RBC, WBC and lymphocyte concentrations.  Linear effect on haugh unit (8 wk) and egg yolk index.  No effect on egg production, egg weight, egg shell breaking strength, egg shell thickness, and yolk color unit. | [1] |
| Weanling pigs | 5 mg  10 mg  15 mg | 35 d (initial BW, 7.21 ± 0.51 kg) | ADG, ADFI, and G:F; DM and nitrogen digestibility; RBC, Hb, Fe, and TIBC; RT, WBC, lymphocyte, IgG, plasma cortisol, IGF-1, TNF-α, and haptoglobin | 5 mg of 5-ALA improved WBC (35 d). 10 mg of 5-ALA increased Hb, Fe and RBC while, 15 mg 5-ALA improved nutrient digestibility (35 d).  Overall no effect on ADG, ADFI, G:F, IgG, TIBC, and lymphocyte.  5-ALA decreased plasma cortisol and TNF-α concentration at 2 hr post-challenge while, 5-ALA reduced IGF-1 (2 hr) and WBC (12 hr) at post-challenge by LPS. No changes in RBC, lymphocyte, and haptoglobin post-challenge by LPS. | [2] |
| Broiler chickens | 5 mg  10 mg  15 mg | 35 d (1-35 d of age) | WG, FI, and FCR; RBC, Hb, Fe, and TIBC; WBC and lymphocyte; weights of liver, spleen, and bursa of Fabricius | Linear improvement of spleen weight and bursa of Fabricius weight. No effect on liver weight.  No differences in WG, FI, FCR, RBC, WBC, lymphocyte, serum Fe, TIBC, and Hb concentrations. | [3] |
| Lactating dairy cows | 10 mg | 14 d (219 ± 36 days in milk) | DMI, milk yield and composition, ECM, MUN, SCC, RBC, Hb, HCT, Fe, and TIBC, as well as WBC, DC, phagocytosis of blood MNC, Con A and PHA-induced proliferation of MNC. | 5-ALA improved milk protein and casein concentration and yields but had no effect on milk yield and other milk parameters.  5-ALA increased the count of WBC, granulocytes, the rate of phagocytosis, and mitogen-induced proliferation of blood MNC.  No effect of 5-ALA on iron status. | [4] |
| Weanling pigs | 500 mg  1 g | 33 d (from 28 d of age) | ADG, ADFI, and G:F; DM, nitrogen and E digestibility; RBC, Hb, HCT, Fe, and TIBC. | 5-ALA increased ADG, G:F, (DM digestibility 33 d), serum Hb, HCT, TIBC, and blood RBC (33 d).  No differences in ADFI, nitrogen, E digestibility, and Fe concentration. | [5] |
| Fattening pigs | 1 g + 20 g OMP | 69 d (initial BW, 49.46 kg) | ADG, ADFI, FCR and feed efficiency; TBARS concentration in loin meat of swine’s. | No effect ADG, ADFI, FCR, and feed efficiency.  Significantly lower TBARS concentration in loin meat of swine’s treated by combined 5-ALA and OMP. | [6] |
| Pregnant and lactating sows | 500 mg  1 g | 42 d (before 14 d of farrowing to weaning) | BW, FI; milk composition; born alive litter size and piglets BW at birth and weaning, ADG; Hb and Fe. | 5-ALA improved milk protein contents, but had no effect on BW, FI, milk fat, and milk lactose.  No effect of 5-ALA supplementation on born alive litter size and piglets BW at birth.  Linear improvement of BW and ADG of piglets of 5-ALA-fed sows.  Linear increase of blood Fe and Hb in piglets of 5-ALA-fed sows. | [7] |
| Weanling pigs | 500 mg | 35 d (from 21 d of age) | ADG, ADFI, and G:F; RBC, Hb, WBC, and DC. | 5-ALA improved RBC. No differences in ADG, ADFI, G:F, Hb, WBC, and DC. | [8] |
| Broiler chickens | Exp 1&2:  10 mg  100 mg  1 g  Exp 3:  10 mg  100 mg  Exp 4:  10 mg | Exp 1, 2&3:  10 d (from 7-17 d of age)  Exp 4:  50 d (from 1-50 d of age) | BWG, FI, and feed efficiency; the expression of T-cell-related mRNA including CD3, IL-2, and IFN –γ in the spleen, as well as phagocytes of blood MNC, Con A and PHA -induced proliferation of splenic MNC. inflammatory response parameters e.g., plasma ceruloplasmin and the mRNA expression levels of IL-2, IL-6, CD3, IFN-γ, iNOS, and TLR as well as TL1A in the spleen as well as RT. Plasma TBARS and spleen weight. | Exp 1&2:  Significantly increased levels of spleen CD3, mitogen (Con A and PHA) induced proliferation of splenic MNC, rates of phagocytosis in blood MNC and plasma TBARS. 5-ALA (1 g) decreased IL-2 levels.  No effect on BWG, FI, spleen weight, and IFN-γ - mRNA expression.  Exp 3:  5-ALA (10 mg) reduced plasma ceruloplasmin concentration (24 hr) and the expression of IFN-γ, iNOS, IL-6, and TL1A mRNA (3 hr) after LPS injection.  No effect on BWG, FI, and RT, levels of CD3, IL-2, TLR2, TLR4, and TLR7 mRNA in the spleen during LPS stimulation.  Exp 4:  5-ALA increased BW, CD3 mRNA expression in the spleen and plasma TBARS concentrations. No changes in feed efficiency. | [9] |
| Weanling pigs | 10 mg  10 mg + 40 mg apramycin | 42 d (from 21 d of age) | ADG, ADFI, and G:F; Hb, HCT, Fe, and TIBC; CD2+, CD4+, CD8+ cells, CD4+ : CD8+, B-cells, MHC-I and II. | 5-ALA alone or in combination with apramycin increased CD8+ cells, B-cells, MHC-I and II.  5-ALA increased serum Hb, HCT, and CD2+ cells.  No differences in ADG, ADFI, G:F, serum Fe, TIBC, CD4+ cells, and the ratio of CD4+ to CD8+ cells. | [10] |
| Laying hens | 5 mg  10 mg  5 mg + 500 mg VC  10 mg + 500 mg VC | 42 d (from 57 wk of age) | ADFI, feed efficiency, egg production and quality e.g., egg (weight, shell breaking strength, shell thickness, shell color, yolk color unit, albumin height, iron concentration in yolk and haugh unit); RBC, Hb, HCT, Fe, and TIBC; WBC and lymphocyte. | 5-ALA alone or in combination with VC increased serum Fe, Hb, RBC count, and raised yolk color unit and Fe concentration in yolk (last 3 wk).  Positive effect of 5-ALA on the color of egg shells (last 3 wk) and the haugh unit (first 3 wk).  5-ALA (10 mg/kg) combined with VC improved egg production (last 3 wk), egg shell thickness (first 3 wk), and lymphocyte count.  No differences in ADFI, feed efficiency, egg weight, egg shell breaking strength, albumin height, HCT, TIBC, and WBC. | [11] |
| Broiler chickens | 5 mg  10 mg  5 mg + 500 mg VC  10 mg + 500 mg VC | 35 d (1-35 d of age) | BWG, FI, FCR, and the mortality rate of the chicks; RBC, Hb, HCT, Fe, and TIBC; WBC and lymphocyte; weights of liver, spleen, thymus, and bursa of Fabricius. | 5-ALA alone or combined (10 mg/kg) with VC improved Fe concentration in serum, liver, and breast meat.  5-ALA (10 mg/kg) combined with VC increased Hb and RBCs count.  No effect on BWG, FI, FCR, mortality rate, WBC, lymphocyte count, HCT, TIBC, and the weight of immune organs. | [12] |
| Pregnant and lactating sows | Exp 1:  90 mg  Exp 2:  90 mg  90 mg + 200 mg Fe dextran | Exp 1:  135 d (during gestation and lactation periods)  Exp 2:  Progeny of sows, 21 d (from birth to wean) | BW, FI; milk composition; born alive litter size and piglets BW at birth and weaning, ADG; Hb, HCT, Fe, and ceruloplasmin oxidase activity. | Exp 1:  Significantly increased plasma and milk concentrations of Hb and Fe.  Significantly increased born alive litter size and piglets BW at birth and weaning.  No changes in BW, FI, milk fat, milk protein, milk lactose and plasma HCT.  Exp 2:  5-ALA alone or with iron injection improved piglet weight (7 & 21 d), ADG (7 d), plasma Fe, Hb and HCT concentration and reduced ceruloplasmin oxidase activity in piglets (7 & 21 d). | [13] |
| Parturient sows | 10 mg  10 mg + 500 mg VC | 28 d (from farrowing to weaning) | BW, ADG, ADFI; milk composition; RBC, Hb, Fe, and TIBC; IgG, WBC and lymphocyte. | Significant increase of RBC, Fe, IgG, milk fat, and protein contents in sows.  Significantly increased Hb and Fe concentrations in piglets of sows treated with 5-ALA.  Positive interaction effect of 5-ALA and VC on the final BW of weaned pigs and ADG.  No differences in ADFI, Hb, TIBC, WBC, lymphocyte, milk glucose, total solid, and Fe concentration in milk of sows.  No differences in RBC, WBC, lymphocyte count, IgG, and TIBC concentrations in piglets. | [14] |
| Weanling pigs | 3 mg  3 mg + 3 mg COS | 35 d (from 21 d of age) | ADG, ADFI, and G:F; DM and nitrogen digestibility; RBC, Hb, Fe, and TIBC; IgG, WBC and lymphocyte. | Positive effect of 5-ALA on RBC, serum Fe, and TIBC.  A synergistic effect of 5-ALA combined with COS on IgG concentration.  No differences in ADG, ADFI, G:F, nutrient digestibility, WBC, lymphocyte count, and Hb. | [15] |
| Laying hens | 2 mg  4 mg | 56 d (from 27 wk of age) | FI, egg production and quality e.g., egg (weight, shell breaking strength, shell thickness, yolk color unit, and haugh unit); RBC, Hb, Fe, and TIBC; WBC and lymphocyte. | Quadratic improvement of WBC and Fe concentrations. Linear and quadratic increase of RBC and lymphocyte counts.  Significant improvement of egg production (first 4 wk), egg weight (last 4 wk), and yolk color unit (last 4 wk), and haugh unit (first 4 wk).  No differences in FI egg shell quality, serum Hb, and TIBC. | [16] |

5-ALA: 5-aminolevulininc acid; ^1^: 5-ALA alone or 5-ALA combination per kg of feed; ^2^: Supplementation period (days); d: days; wk: weeks; RBC: Red blood cells; Hb: Hemoglobin; Fe: Iron; TIBC: Total iron binding capacity; WBC: White blood cells; BW: Body weight; ADG: Average daily gain; ADFI: Average daily feed intake; G:F: Gain:feed ratio; DM: Dry matter; RT: Rectal temperature; IgG: Immunoglobin G; IGF-1: Insulin-like growth factor-1; TNF-α: Tumor necrosis factor-α; hr: Hours; LPS: *Escherichia coli* lipopolysaccharide; WG: Weight gain; FI: Feed intake; FCR: Feed conversion ratio; DMI: Dry matter intake; ECM: Energy-corrected milk; MUN: Milk urea nitrogene; SCC: Somatic cell count; HCT: Hematocrit; DC: Differential count of WBC; MNC: Mononuclear cells; Con A: Concanavalin A; PHA: Phytohemagglutinin; E: Energy; OMP: Oriental medicinal plants; TBARS: Thiobarbituric acid reactive substances; BWG: Body weight gain; CD3: Cluster of differentiation 3; IL: Interleukin; IFN-γ: Interferon-γ; iNOS: Inducible nitric oxide synthase; TLR: Toll-like receptor; TL1A: Tumor necrosis factor-like ligand 1A; CD2+, CD4+, and CD8+: Cluster of differentiation antigens positive cells 2, 4, and 8; MHC-I and II: Major histocompatibility complex class I and II; VC: Vitamin C; COS: Chito-oligosaccharide.

References

1. Chen, Y.J.; Cho, J.H.; Yoo, J.S.; Wang, Y.; Huang, Y.; Kim, I.H. Evaluation of δ-aminolevulinic acid on serum iron status, blood characteristics, egg performance and quality in laying hens. *Asian-Australasian Journal of Animal Sciences* **2008**, *21*, 1355-1360, doi:<https://doi.org/10.5713/ajas.2008.70634>.

2. Chen, Y.J.; Kim, I.H.; Cho, J.H.; Min, B.J.; Yoo, J.S.; Wang, Q. Effect of δ-aminolevulinic acid on growth performance, nutrient digestibility, blood parameters and the immune response of weanling pigs challenged with Escherichia coli lipopolysaccharide. *Livestock Science* **2008**, *114*, 108-116, doi:<https://doi.org/10.1016/j.livsci.2007.04.015>.

3. Chen, Y.J.; Kim, I.H.; Cho, J.H.; Yoo, J.S.; Kim, H.J.; Shin, S.O. Utilization of δ-aminolevulinic acid for livestock: Blood characteristics and immune organ weight in broilers. *Journal of Animal and Feed Sciences* **2008**, *17*, 215-223, doi:<https://doi.org/10.22358/jafs/66601/2008>.

4. Hendawy, A.O.; Shiraishi, M.; Takeya, H.; Sugimura, S.; Miyanari, S.; Taniguchi, S.; Sato, K. Effects of 5-aminolevulinic acid supplementation on milk production, iron status, and immune response of dairy cows. *J. Dairy Sci.* **2019**, *102*, 11009-11015, doi:<https://doi.org/10.3168/jds.2018-15982>.

5. Hossain, M.M.; Park, J.W.; Kim, I.H. δ-Aminolevulinic acid, and lactulose supplements in weaned piglets diet: Effects on performance, fecal microbiota, and in-vitro noxious gas emissions. *Livestock Science* **2016**, *183*, 84-91, doi:<https://doi.org/10.1016/j.livsci.2015.11.021>.

6. Kang, S.N.; Chu, G.M.; Song, Y.M.; Jin, S.K.; Hwang, I.H.; Kim, I.S. The effects of replacement of antibiotics with by-products of oriental medicinal plants on growth performance and meat qualities in fattening pigs. *Animal Science Journal* **2012**, *83*, 245-251, doi:<https://doi.org/10.1111/j.1740-0929.2011.00942.x>.

7. Lee, S.I.; Li, T.S.; Kim, I.H. Dietary supplementation of delta-aminolevulinic acid to lactating sows improves growth performance and concentration of iron and hemoglobin of suckling piglets. *Indian Journal of Animal Sciences* **2016**, *86*, 781-785.

8. Mateo, R.D.; Morrow, J.L.; Dailey, J.W.; Ji, F.; Kim, S.W. Use of δ-aminolevulinic acid in swine diet: Effect on growth performance, behavioral characteristics and hematological/immune status in nursery pigs. *Asian-Australian Journal of Animal Sciences* **2006**, *19*, 97-101, doi:<https://doi.org/10.5713/ajas.2006.97>.

9. Sato, K.; Matsushita, K.; Takahashi, K.; Aoki, M.; Fuziwara, J.; Miyanari, S.; Kamada, T. Dietary supplementation with 5-aminolevulinic acid modulates growth performance and inflammatory responses in broiler chickens. *Poultry science* **2012**, *91*, 1582-1589, doi:<https://doi.org/10.3382/ps.2010-01201>.

10. Wang, J.P.; Jung, J.H.; Kim, I.H. Effects of dietary supplementation with delta-aminolevulinic acid on growth performance, hematological status, and immune responses of weanling pigs. *Livestock Science* **2011**, *140*, 131-135, doi:<https://doi.org/10.1016/j.livsci.2011.02.017>.

11. Wang, J.P.; Lee, J.H.; Jang, H.D.; Yan, L.; Cho, J.H.; Kim, I.H. Effects of δ-aminolevulinic acid and vitamin C supplementation on iron status, production performance, blood characteristics and egg quality of laying hens. *Journal of Animal Physiology and Animal Nutrition* **2011**, *95*, 417-423, doi:<https://doi.org/10.1111/j.1439-0396.2010.01067.x>.

12. Wang, J.P.; Yan, L.; Lee, J.H.; Zhou, T.X.; Kim, I.H. Effects of dietary delta-aminolevulinic acid and vitamin C on growth performance, immune organ weight and ferrum status in broiler chicks. *Livestock Science* **2011**, *135*, 148-152, doi:<https://doi.org/10.1016/j.livsci.2010.06.161>.

13. Wang, J.P.; Kim, I.H. Effects of iron injection at birth on neonatal iron status in young pigs from first-parity sows fed delta-aminolevulinic acid. *Animal Feed Science and Technology* **2012**, *178*, 151-157, doi:<https://doi.org/10.1016/j.anifeedsci.2012.08.011>.

14. Wang, J.P.; Kim, H.J.; Chen, Y.J.; Yoo, J.S.; Cho, J.H.; Kang, D.K.; Hyun, Y.; Kim, I.H. Effects of delta-aminolevulinic acid and vitamin C supplementation on feed intake, backfat, and iron status in sows. *J. Anim. Sci.* **2009**, *87*, 3589-3595, doi:<https://doi.org/10.2527/jas.2008-1489>.

15. Yan, L.; Kim, I.H. Evaluation of dietary supplementation of delta-aminolevulinic acid and chitooligosaccharide on growth performance, nutrient digestibility, blood characteristics, and fecal microbial shedding in weaned pigs. *Animal Feed Science and Technology* **2011**, *169*, 275-280, doi:<https://doi.org/10.1016/j.anifeedsci.2011.06.017>.

16. Yan, L.; Lee, J.H.; Meng, Q.W.; Ao, X.; Kim, I.H. Evaluation of dietary supplementation of delta-aminolevulinic acid and chito-oligosaccharide on production performance, egg quality and hematological characteristics in laying hens. *Asian-Australian journal of animal sciences* **2010**, *23*, 1028-1033, doi:<https://doi.org/10.5713/ajas.2010.90639>.
