# Supplementary material for: Effects of 5-Aminolevulinic Acid as a Supplement on Animal Performance, Iron Status, and Immune Response in Farm Animals: A Review
Source: Animals (Basel). 2020 Aug 4;10(8):1352. doi: 10.3390/ani10081352 (PMC7459508; doi:10.3390/ani10081352)
Supplement: Supplementary file 1 [file animals-10-01352-s001.zip › Supplementary Files/Table S2.docx]

**Table S2.** Evaluation of the quality of the papers according to the selection criteria.

| **Author** | **A** | **B** | **C** | **D** | **E** | **F** | **G** | **Total** |
| --- | --- | --- | --- | --- | --- | --- | --- | --- |
| Chen*, et al.* [1] | 2 | 1 | 1 | 1 | 2 | 2 | 2 | 11 |
| Chen*, et al.* [2] | 2 | 1 | 1 | 1 | 2 | 1 | 2 | 10 |
| Chen*, et al.* [3] | 2 | 1 | 1 | 1 | 2 | 2 | 2 | 11 |
| Hendawy*, et al.* [4] | 2 | 1 | 1 | 1 | 2 | 1 | 2 | 10 |
| Hossain*, et al.* [5] | 2 | 1 | 1 | 1 | 2 | 2 | 2 | 11 |
| Kang*, et al.* [6] | 2 | 1 | 1 | 1 | 0 | 1 | 2 | 8 |
| Lee*, et al.* [7] | 2 | 1 | 1 | 1 | 2 | 1 | 2 | 10 |
| Mateo*, et al.* [8] | 0 | 1 | 1 | 1 | 2 | 2 | 2 | 9 |
| Sato*, et al.* [9] | 2 | 1 | 1 | 1 | 2 | 1 | 2 | 10 |
| Wang*, et al.* [10] | 2 | 1 | 1 | 1 | 2 | 2 | 2 | 11 |
| Wang*, et al.* [11] | 2 | 1 | 1 | 1 | 2 | 1 | 2 | 10 |
| Wang*, et al.* [12] | 2 | 1 | 1 | 1 | 2 | 2 | 2 | 11 |
| Wang and Kim [13] | 2 | 1 | 1 | 1 | 2 | 1 | 2 | 10 |
| Wang*, et al.* [14] | 2 | 1 | 1 | 1 | 0 | 1 | 2 | 8 |
| Yan and Kim [15] | 2 | 1 | 1 | 1 | 2 | 1 | 2 | 10 |
| Yan*, et al.* [16] | 2 | 1 | 1 | 1 | 2 | 2 | 2 | 11 |

A. Randomization: 2 for randomized experiments, 1 for unclear and 0 for non-randomized experiments.

B. Allocation concealment: trials that described procedures to conceal allocation obtained a score of 2 while when the procedure was not reported obtained a score of 1 when allocation was not concealed trials obtained a a score of 0.

C. Performance bias: when treatment was conducted by an experimenter who was blinded to the study aim/hypothesis the study obtained a score of 2, but when blinding of the experimenter was not reported it received a score of 1 when experimenters were not blinded to the study aim/hypothesis the study obtained a score of 0.

D. Detection bias: studies that performed blind evaluation by a blinded examiner obtained a score of 2, but when not clearly reported these studies received a score of 1 when examiners were not blinded to the study aim/hypothesis the studies received a score of 0.

E. Reporting bias: when all outcomes described in the methods section were reported in the results the study received a score of 2, studies with missing outcomes obtained a score of 1 whereas studies with reports that were inconsistent between the abstract, results and discussion received a score of 0.

F. Sample size: 2 for trials using more than 30 pigs or 50 birds per treatment and 1 for those using 30 pigs, 50 birds or less.

G. Breed or genetic line: when breed or genetic line was detailed, trials obtained a score of 2, but when not described trials received a score of 1.

References

1. Chen, Y.J.; Cho, J.H.; Yoo, J.S.; Wang, Y.; Huang, Y.; Kim, I.H. Evaluation of δ-aminolevulinic acid on serum iron status, blood characteristics, egg performance and quality in laying hens. *Asian-Australasian Journal of Animal Sciences* **2008**, *21*, 1355-1360, doi:<https://doi.org/10.5713/ajas.2008.70634>.

2. Chen, Y.J.; Kim, I.H.; Cho, J.H.; Min, B.J.; Yoo, J.S.; Wang, Q. Effect of δ-aminolevulinic acid on growth performance, nutrient digestibility, blood parameters and the immune response of weanling pigs challenged with Escherichia coli lipopolysaccharide. *Livestock Science* **2008**, *114*, 108-116, doi:<https://doi.org/10.1016/j.livsci.2007.04.015>.

3. Chen, Y.J.; Kim, I.H.; Cho, J.H.; Yoo, J.S.; Kim, H.J.; Shin, S.O. Utilization of δ-aminolevulinic acid for livestock: Blood characteristics and immune organ weight in broilers. *Journal of Animal and Feed Sciences* **2008**, *17*, 215-223, doi:<https://doi.org/10.22358/jafs/66601/2008>.

4. Hendawy, A.O.; Shiraishi, M.; Takeya, H.; Sugimura, S.; Miyanari, S.; Taniguchi, S.; Sato, K. Effects of 5-aminolevulinic acid supplementation on milk production, iron status, and immune response of dairy cows. *J. Dairy Sci.* **2019**, *102*, 11009-11015, doi:<https://doi.org/10.3168/jds.2018-15982>.

5. Hossain, M.M.; Park, J.W.; Kim, I.H. δ-Aminolevulinic acid, and lactulose supplements in weaned piglets diet: Effects on performance, fecal microbiota, and in-vitro noxious gas emissions. *Livestock Science* **2016**, *183*, 84-91, doi:<https://doi.org/10.1016/j.livsci.2015.11.021>.

6. Kang, S.N.; Chu, G.M.; Song, Y.M.; Jin, S.K.; Hwang, I.H.; Kim, I.S. The effects of replacement of antibiotics with by-products of oriental medicinal plants on growth performance and meat qualities in fattening pigs. *Animal Science Journal* **2012**, *83*, 245-251, doi:<https://doi.org/10.1111/j.1740-0929.2011.00942.x>.

7. Lee, S.I.; Li, T.S.; Kim, I.H. Dietary supplementation of delta-aminolevulinic acid to lactating sows improves growth performance and concentration of iron and hemoglobin of suckling piglets. *Indian Journal of Animal Sciences* **2016**, *86*, 781-785.

8. Mateo, R.D.; Morrow, J.L.; Dailey, J.W.; Ji, F.; Kim, S.W. Use of δ-aminolevulinic acid in swine diet: Effect on growth performance, behavioral characteristics and hematological/immune status in nursery pigs. *Asian-Australian Journal of Animal Sciences* **2006**, *19*, 97-101, doi:<https://doi.org/10.5713/ajas.2006.97>.

9. Sato, K.; Matsushita, K.; Takahashi, K.; Aoki, M.; Fuziwara, J.; Miyanari, S.; Kamada, T. Dietary supplementation with 5-aminolevulinic acid modulates growth performance and inflammatory responses in broiler chickens. *Poultry science* **2012**, *91*, 1582-1589, doi:<https://doi.org/10.3382/ps.2010-01201>.

10. Wang, J.P.; Jung, J.H.; Kim, I.H. Effects of dietary supplementation with delta-aminolevulinic acid on growth performance, hematological status, and immune responses of weanling pigs. *Livestock Science* **2011**, *140*, 131-135, doi:<https://doi.org/10.1016/j.livsci.2011.02.017>.

11. Wang, J.P.; Lee, J.H.; Jang, H.D.; Yan, L.; Cho, J.H.; Kim, I.H. Effects of δ-aminolevulinic acid and vitamin C supplementation on iron status, production performance, blood characteristics and egg quality of laying hens. *Journal of Animal Physiology and Animal Nutrition* **2011**, *95*, 417-423, doi:<https://doi.org/10.1111/j.1439-0396.2010.01067.x>.

12. Wang, J.P.; Yan, L.; Lee, J.H.; Zhou, T.X.; Kim, I.H. Effects of dietary delta-aminolevulinic acid and vitamin C on growth performance, immune organ weight and ferrum status in broiler chicks. *Livestock Science* **2011**, *135*, 148-152, doi:<https://doi.org/10.1016/j.livsci.2010.06.161>.

13. Wang, J.P.; Kim, I.H. Effects of iron injection at birth on neonatal iron status in young pigs from first-parity sows fed delta-aminolevulinic acid. *Animal Feed Science and Technology* **2012**, *178*, 151-157, doi:<https://doi.org/10.1016/j.anifeedsci.2012.08.011>.

14. Wang, J.P.; Kim, H.J.; Chen, Y.J.; Yoo, J.S.; Cho, J.H.; Kang, D.K.; Hyun, Y.; Kim, I.H. Effects of delta-aminolevulinic acid and vitamin C supplementation on feed intake, backfat, and iron status in sows. *J. Anim. Sci.* **2009**, *87*, 3589-3595, doi:<https://doi.org/10.2527/jas.2008-1489>.

15. Yan, L.; Kim, I.H. Evaluation of dietary supplementation of delta-aminolevulinic acid and chitooligosaccharide on growth performance, nutrient digestibility, blood characteristics, and fecal microbial shedding in weaned pigs. *Animal Feed Science and Technology* **2011**, *169*, 275-280, doi:<https://doi.org/10.1016/j.anifeedsci.2011.06.017>.

16. Yan, L.; Lee, J.H.; Meng, Q.W.; Ao, X.; Kim, I.H. Evaluation of dietary supplementation of delta-aminolevulinic acid and chito-oligosaccharide on production performance, egg quality and hematological characteristics in laying hens. *Asian-Australian journal of animal sciences* **2010**, *23*, 1028-1033, doi:<https://doi.org/10.5713/ajas.2010.90639>.
