# Supplementary material for: Effects of 5-Aminolevulinic Acid as a Supplement on Animal Performance, Iron Status, and Immune Response in Farm Animals: A Review
Source: Animals (Basel). 2020 Aug 4;10(8):1352. doi: 10.3390/ani10081352 (PMC7459508; doi:10.3390/ani10081352)
Supplement: Supplementary file 1 [file animals-10-01352-s001.zip › Supplementary Files/Table S3.docx]

**Table S3.** Effects of 5-aminolevulininc acid compared with other alternative treatments and 5-aminolevulininc acid combination with other treatments.

| **Item** | **ALA** | **VC** | **ALA × VC** | **ALA** | **COS** | **ALA × COS** | **ALA** | **ANTI** | **ALA ×**  **ANTI** | **ALA** | **Fe** | **ALA**  **× Fe** |
| --- | --- | --- | --- | --- | --- | --- | --- | --- | --- | --- | --- | --- |
| Broiler chickens | | | | | | | | | | | | |
| BWG (g) | 0 | 0 | 0 | ND | ND | ND | ND | ND | ND | ND | ND | ND |
| FI (g) | 0 | 0 | 0 | ND | ND | ND | ND | ND | ND | ND | ND | ND |
| FCR | 0 | 0 | 0 | ND | ND | ND | ND | ND | ND | ND | ND | ND |
| Hemoglobin (g/dL) | 0 | 0 | + | ND | ND | ND | ND | ND | ND | ND | ND | ND |
| Fe (μg/dL) | + | 0 | + | ND | ND | ND | ND | ND | ND | ND | ND | ND |
| TIBC (μg/dL) | 0 | 0 | 0 | ND | ND | ND | ND | ND | ND | ND | ND | ND |
| Fe in liver (mg kg^−1^) | + | + | + | ND | ND | ND | ND | ND | ND | ND | ND | ND |
| Fe in breast meat (mg kg^−1^) | + | + | + | ND | ND | ND | ND | ND | ND | ND | ND | ND |
| RBC (×10^6^/mm^3^) | + | 0 | 0 | ND | ND | ND | ND | ND | ND | ND | ND | ND |
| Hematocrit (%) | 0 | + | 0 | ND | ND | ND | ND | ND | ND | ND | ND | ND |
| WBC (×10^4^/mm^3^) | 0 | 0 | 0 | ND | ND | ND | ND | ND | ND | ND | ND | ND |
| Lymphocyte (%) | 0 | 0 | 0 | ND | ND | ND | ND | ND | ND | ND | ND | ND |
| Organ weight (g/100 g BW): | | | | | | | | | | | | |
| Liver | 0 | 0 | 0 | ND | ND | ND | ND | ND | ND | ND | ND | ND |
| Spleen | 0 | 0 | 0 | ND | ND | ND | ND | ND | ND | ND | ND | ND |
| Bursa of Fabricius | 0 | 0 | 0 | ND | ND | ND | ND | ND | ND | ND | ND | ND |
| Thymus | 0 | 0 | 0 | ND | ND | ND | ND | ND | ND | ND | ND | ND |
| Laying hens | | | | | | | | | | | | |
| Haemoglobin (g/dl) | + | 0 | + | ND | ND | ND | ND | ND | ND | ND | ND | ND |
| Haematocrit (%) | 0 | 0 | 0 | ND | ND | ND | ND | ND | ND | ND | ND | ND |
| Fe (µg/dl) | + | 0 | + | ND | ND | ND | ND | ND | ND | ND | ND | ND |
| TIBC (µg/dl) | 0 | 0 | 0 | ND | ND | ND | ND | ND | ND | ND | ND | ND |
| RBC (10^6^/mm^3^) | + | 0 | + | ND | ND | ND | ND | ND | ND | ND | ND | ND |
| WBC (10^4^/mm^3^) | 0 | 0 | 0 | ND | ND | ND | ND | ND | ND | ND | ND | ND |
| Lymphocyte (%) | 0 | 0 | + | ND | ND | ND | ND | ND | ND | ND | ND | ND |
| ADFI (g) | 0 | 0 | 0 | ND | ND | ND | ND | ND | ND | ND | ND | ND |
| Feed efficiency | 0 | 0 | 0 | ND | ND | ND | ND | ND | ND | ND | ND | ND |
| Egg production (%) | 0 | 0 | 0 | ND | ND | ND | ND | ND | ND | ND | ND | ND |
| Egg weight (g) | 0 | 0 | 0 | ND | ND | ND | ND | ND | ND | ND | ND | ND |
| Egg shell breaking strength (kg/cm^2^) at 3 weeks | 0 | 0 | 0 | ND | ND | ND | ND | ND | ND | ND | ND | ND |
| Egg shell breaking strength (kg/cm^2^) at 6 weeks | 0 | 0 | 0 | ND | ND | ND | ND | ND | ND | ND | ND | ND |
| Egg shell thickness (10^-2^ mm) at 3 weeks | 0 | 0 | + | ND | ND | ND | ND | ND | ND | ND | ND | ND |
| Egg shell thickness (10^-2^ mm) at 6 weeks | 0 | 0 | 0 | ND | ND | ND | ND | ND | ND | ND | ND | ND |
| Yolk colour unit at 3 weeks | 0 | 0 | 0 | ND | ND | ND | ND | ND | ND | ND | ND | ND |
| Yolk colour unit at 6 weeks | + | + | 0 | ND | ND | ND | ND | ND | ND | ND | ND | ND |
| Egg shell colour at 3 weeks | 0 | + | 0 | ND | ND | ND | ND | ND | ND | ND | ND | ND |
| Egg shell colour at 6 weeks | + | 0 | 0 | ND | ND | ND | ND | ND | ND | ND | ND | ND |
| Albumin height (mm) at 3 weeks | 0 | 0 | 0 | ND | ND | ND | ND | ND | ND | ND | ND | ND |
| Albumin height (mm) at 6 weeks | + | 0 | 0 | ND | ND | ND | ND | ND | ND | ND | ND | ND |
| Haugh unit at 3 weeks | + | 0 | 0 | ND | ND | ND | ND | ND | ND | ND | ND | ND |
| Haugh unit at 6 weeks | 0 | + | 0 | ND | ND | ND | ND | ND | ND | ND | ND | ND |
| Fe concentration in yolk (mg/kg) at 6 weeks | 0 | 0 | + | ND | ND | ND | ND | ND | ND | ND | ND | ND |
| Weanling pigs | | | | | | | | | | | | |
| BW (kg) | ND | ND | ND | 0 | 0 | 0 | 0 | 0 | 0 | ++ | + | ++ |
| ADG (g) | ND | ND | ND | 0 | 0 | 0 | 0 | 0 | 0 | 0 | 0 | 0 |
| ADFI (g) | ND | ND | ND | 0 | 0 | 0 | 0 | 0 | 0 | ND | ND | ND |
| G:F | ND | ND | ND | 0 | 0 | 0 | 0 | 0 | 0 | ND | ND | ND |
| DM digestibility | ND | ND | ND | 0 | 0 | 0 | ND | ND | ND | ND | ND | ND |
| Nitrogen digestibility | ND | ND | ND | 0 | 0 | 0 | ND | ND | ND | ND | ND | ND |
| Fe (mg/L) | ND | ND | ND | + | 0 | 0 | 0 | 0 | 0 | + | + | ++ |
| TIBC (mg/L) | ND | ND | ND | + | 0 | 0 | 0 | 0 | 0 | ND | ND | ND |
| Haemoglobin (g/L) | ND | ND | ND | 0 | 0 | 0 | + | 0 | 0 | + | ++ | ++ |
| Haematocrit (%) | ND | ND | ND | ND | ND | ND | + | 0 | 0 | + | ++ | ++ |
| RBC (×10^6^/mm^3^) | ND | ND | ND | + | 0 | 0 | ND | ND | ND | ND | ND | ND |
| CPOA, IU/mL | ND | ND | ND | ND | ND | ND | ND | ND | ND | + | + | ++ |
| WBC (×10^3^/mm^3^) | ND | ND | ND | 0 | 0 | 0 | ND | ND | ND | ND | ND | ND |
| Lymphocyte (%) | ND | ND | ND | 0 | + | 0 | ND | ND | ND | ND | ND | ND |
| IgG (g/L) | ND | ND | ND | 0 | 0 | + | ND | ND | ND | ND | ND | ND |
| CD2+ | ND | ND | ND | ND | ND | ND | + | 0 | 0 | ND | ND | ND |
| CD4+ | ND | ND | ND | ND | ND | ND | 0 | 0 | 0 | ND | ND | ND |
| CD8+ | ND | ND | ND | ND | ND | ND | + | 0 | + | ND | ND | ND |
| CD4+:CD8+ | ND | ND | ND | ND | ND | ND | 0 | 0 | 0 | ND | ND | ND |
| B-cells | ND | ND | ND | ND | ND | ND | + | 0 | + | ND | ND | ND |
| MHC-I | ND | ND | ND | ND | ND | ND | + | 0 | + | ND | ND | ND |
| MHC-II | ND | ND | ND | ND | ND | ND | + | 0 | + | ND | ND | ND |
| Parturient sows | | | | | | | | | | | | |
| ADFI (kg) | 0 | 0 | 0 | ND | ND | ND | ND | ND | ND | ND | ND | ND |
| IgG (mg/kg) | + | 0 | + | ND | ND | ND | ND | ND | ND | ND | ND | ND |
| RBC (×10^6^/mL) | + | 0 | 0 | ND | ND | ND | ND | ND | ND | ND | ND | ND |
| WBC (×10^3^/mL) | 0 | 0 | 0 | ND | ND | ND | ND | ND | ND | ND | ND | ND |
| Lymphocyte (%) | 0 | 0 | 0 | ND | ND | ND | ND | ND | ND | ND | ND | ND |
| Haemoglobin (g/dL) | 0 | 0 | 0 | ND | ND | ND | ND | ND | ND | ND | ND | ND |
| Fe (μg/dL) | + | 0 | 0 | ND | ND | ND | ND | ND | ND | ND | ND | ND |
| TIBC (μg/dL) | 0 | 0 | 0 | ND | ND | ND | ND | ND | ND | ND | ND | ND |
| Milk fat (%) | + | 0 | 0 | ND | ND | ND | ND | ND | ND | ND | ND | ND |
| Milk protein (%) | + | 0 | 0 | ND | ND | ND | ND | ND | ND | ND | ND | ND |
| Milk glucose (mg/dL) | 0 | 0 | 0 | ND | ND | ND | ND | ND | ND | ND | ND | ND |
| Milk Solid (%) | 0 | 0 | 0 | ND | ND | ND | ND | ND | ND | ND | ND | ND |
| Milk Fe (mg/L) | + | 0 | 0 | ND | ND | ND | ND | ND | ND | ND | ND | ND |
| Final BW of piglets (kg) | 0 | 0 | + | ND | ND | ND | ND | ND | ND | ND | ND | ND |
| ADG of piglets (kg) | 0 | 0 | + | ND | ND | ND | ND | ND | ND | ND | ND | ND |
| IgG of piglets (mg/dL) | 0 | + | 0 | ND | ND | ND | ND | ND | ND | ND | ND | ND |
| RBC of piglets (×10^6^/mL) | 0 | 0 | 0 | ND | ND | ND | ND | ND | ND | ND | ND | ND |
| Haemoglobin of piglets (g/dL) | + | 0 | 0 | ND | ND | ND | ND | ND | ND | ND | ND | ND |
| Fe of piglets (μg/dL) | + | 0 | 0 | ND | ND | ND | ND | ND | ND | ND | ND | ND |
| WBC of piglets (×10^3^/mL) | 0 | 0 | 0 | ND | ND | ND | ND | ND | ND | ND | ND | ND |
| Lymphocyte of piglets (%) | 0 | 0 | 0 | ND | ND | ND | ND | ND | ND | ND | ND | ND |

ALA: 5-aminolevulininc acid; VC: Vitamin C; ALA × VC: 5-aminolevulininc acid and vitamin C interaction; COS: Chito-oligosaccharide; ALA × COS: 5-aminolevulininc acid and chito-oligosaccharide interaction; ANTI: Antibiotics (apramycin); ALA × ANTI: 5-aminolevulininc acid and antibiotics interaction; Fe: Iron injection (iron dextran); ALA × Fe: 5-aminolevulininc acid and iron injection interaction; +: Positive effect; 0: No effect; -: Negative effect; ND: Not determined; BWG: Body weight gain; FI: Feed intake; FCR: Feed conversion ratio; TIBC: Total Fe binding capacity; RBC: Red blood cells; WBC: White blood cells; BW: Body weight; ADFI: Average daily feed intake; ADG: Average daily gain; G:F: gain:feed; DM: Dry matter; CPOA: Ceruloplasmin oxidase activity; IgG: Immunoglobin G; CD2+, CD4+, and CD8+: Cluster of differentiation antigens positive cells 2, 4, and 8; MHC-I and II: Major histocompatibility complex class I and II.
